# Human Milk Oligosaccharides Exhibit Biofilm Eradication Activity Against Matured Biofilms Formed by Different Pathogen Species

**DOI:** 10.3389/fmicb.2021.794441

**Published:** 2022-01-05

**Authors:** Sylwia Jarzynka, Riccardo Spott, Tinatini Tchatchiashvili, Nico Ueberschaar, Mark Grevsen Martinet, Kamila Strom, Tomasz Kryczka, Aleksandra Wesołowska, Mathias W. Pletz, Gabriela Olędzka, Oliwia Makarewicz

**Affiliations:** ^1^Department of Medical Biology, Medical University of Warsaw, Warsaw, Poland; ^2^Institute of Infectious Diseases and Infection Control, Jena University Hospital, Jena, Germany; ^3^IncfectoGnostics Research Campus, Friedrich Schiller University Jena, Jena, Germany; ^4^Mass Spectrometry Platform, Friedrich Schiller University Jena, Jena, Germany; ^5^Department of Development of Nursing, Social and Medical Sciences, Medical University of Warsaw, Warsaw, Poland; ^6^Department of Medical Biology, Laboratory of Human Milk and Lactation Research at Regional Human Milk Bank in Holy Family Hospital, Medical University of Warsaw, Warsaw, Poland

**Keywords:** biofilms, infectious diseases, enterococci, fucosyllactose, lactose

## Abstract

Human milk oligosaccharides (HMOs) have been shown to exhibit plenty of benefits for infants, such as prebiotic activity shaping the gut microbiota and immunomodulatory and anti-inflammatory activity. For some pathogenic bacteria, antimicrobial activity has been proved, but most studies focus on group B streptococci. In the present study, we investigated the antimicrobial and antibiofilm activities of the total and fractionated HMOs from pooled human milk against four common human pathogenic Gram-negative species (*Klebsiella pneumoniae*, *Acinetobacter baumannii*, *Pseudomonas aeruginosa*, and *Burkholderia cenocepacia*) and three Gram-positive species (*Staphylococcus aureus*, *Enterococcus faecium*, and *Enterococcus faecalis*). The activity of HMOs against enterococci and *B. cenocepacia* are addressed here for the first time. We showed that HMOs exhibit a predominant activity against the Gram-positive species, with *E. faecalis* being the most sensitive to the HMOs, both in planktonic bacteria and in biofilms. In further tests, we could exclude fucosyllactose as the antibacterial component. The biological significance of these findings may lie in the prevention of skin infections of the mother’s breast as a consequence of breastfeeding-induced skin laceration and/or protection of the infants’ nasopharynx and lung from respiratory pathogens such as staphylococci.

## Introduction

Human milk is a unique fluid composed of lactose, lipids, proteins, and free oligosaccharides. Human milk oligosaccharides (HMOs) being the third-largest fraction (after lactose and fats) of organic compounds form the biocidal group with the most potential that might be of therapeutic interest (Bode, 2015). The concentration of HMOs ranges between 5 and 23 g/L and exceeds the concentration of oligosaccharides in bovine milk by 100–1,000-fold (Bode, 2015). HMOs pass the stomach undigested into the intestinal tract, where they act as prebiotics shaping the gut microbiome of fed infants by promoting the growth of beneficial species such as bifidobacteria while inhibiting adhesion of pathogenic bacteria, fungi, or viruses to the epithelial cells (Bode, 2015). They can be also determined in infants’ urine and circulate systemically in high concentrations (Rudloff et al., 2012). It is supposed that they reach almost all organs including the brain and the lung. They have been shown to exhibit immunomodulatory activity by reducing the pro-inflammatory cytokines (Eiwegger et al., 2010; Ackerman et al., 2017) and by reprogramming epithelial cells, making them more resistant to invasion by pathogenic species (Bode, 2015).

The prebiotic and immunomodulatory activity depends on the structure of the HMOs. More than 200 individual structures were identified by mass spectrometry (MS) so far, and the composition strongly varies between women (Bode, 2015). In general, the backbone of the HMOs is composed of 3–15 monosaccharide units of galactose (Gal), glucose (Glc), and *N*-acetyl-glucosamine (GlcNAc) that can be linked β1-4, β1-3, or β1-6, forming linear and branched chains. These can be further modified by fucose (Fuc) and *N*-acetylneuraminic acid (Neu5Ac). Fucosylation is achieved by fucosyltransferase 2 (FUT2, α1-2 linkage to Gal) and/or fucosyltransferase 3 (FUC3, α1-3/4 linkage to Gal); both are also responsible for the fucosylation of the Lewis blood group epitopes. Neu5Ac is added to the backbone of the HMOs in an α2-3 or α2-6 manner to Gal or GlcNAc via different sialyltransferases (SIAT).

The most active HMOs are supposed to be those fucosylated and sialylated variants, but antimicrobial and antibiofilm activities have been demonstrated primarily against *Streptococcus agalactiae*, the leading causatives of invasive bacterial infections in newborns, particularly for the neutral fraction of pooled HMOs (Ackerman et al., 2017) of selected donors. Due to their bioactivity, we hypothesized that HMOs might exhibit antimicrobial activity against a wide spectrum of human pathogenic bacteria, both against planktonic bacteria and also against their biofilms.

Biofilms represent the preferred lifestyle of microorganisms and are present in more than 80% of all human bacterial infections (Romling and Balsalobre, 2012). Compared to their planktonic counterparts, biofilm-embedded bacteria show up to 1,000-fold higher resistance to antibiotics (Cosgrove et al., 2002) due to the protective biofilm matrix properties and metabolic dormancy (Lewis, 2001). Thus, under therapeutically feasible antibiotic concentrations, biofilms remain robust, leading to treatment failure and recurring infections. Therefore, novel substances with potential antibacterial and antibiofilm activities are being sought.

As HMOs are known to exhibit probiotic activity shaping the gut of the newborns (Kunz, 2012; Bode, 2018), their activity against the most common human pathogens was investigated.

Therefore, we investigated the antibiofilm activities of the total and fractionated HMOs from pooled human milk of nine donors after extraction of the saccharide fraction against common human pathogenic Gram-negative (*Klebsiella pneumoniae*, *Acinetobacter baumannii*, *Pseudomonas aeruginosa*, and *Burkholderia cenocepacia*) and Gram-positive species (*Staphylococcus aureus*, *Enterococcus faecium*, and *Enterococcus faecalis*).

## Materials and Methods

### Participants and Milk Collection

This study was approved by the ethics committee of WUM in October 2018 under the registry number AKBE/180/2018 followed by an approval by the ethics committee of JUH in April 2019 under the registry number 2019–1360-Material. Human milk was obtained from nine healthy donors, who signed written informed consent, by the Human Milk Bank in Warsaw (Poland) between 05.03.2018 and 26.08.2018. Donors were in the initial period of lactation (within the first year). Milk samples of 400 ml from each donor (a total of 3,600 ml) were collected by the participants and frozen in a standard freezer after collection until they were picked by a service of the Human Milk Bank. The milk was stored at −20°C until processing.

### Isolation of the Carbohydrate Fraction From Human Milk

The milk samples were polled after thawing, and skimmed milk was obtained by centrifugation at 3,000 *g* and 4°C for 20 min in two repetitions. The proteins were precipitated by the addition of 96% ethanol to the supernatant (1:1) and incubation at 4°C overnight followed by centrifugation at 3,000 g and 4°C for 30 min. The ethanol in the supernatant that contained an enriched carbohydrate fraction was evaporated by a rotary evaporator at approximately 80°C (Laborota 4001, Heidolph Instruments, Schwabach, Germany). The total saccharides hereinafter referred to as HMOs were finally freeze-dried, and the powder was stored at 4°C. Based on the preliminary works, it can be expected that after the extraction, the oligosaccharide fraction contains primary lactose and approximately 7–21% of HMOs (Bode, 2012).

The lactose was enzymatically digested by adding 100 U of β-D-galactoside galactohydrolase (lactase F) (Sigma-Aldrich, St. Louis, United States) to 100 mg of lyophilized HMOs resolved in 1 ml of deionized water and subsequently incubating overnight at 37°C. The digested HMOs were freeze-dried thereafter and stored at 4°C.

### Bacterial Strains

Different clinical isolates (provided by Jena University Hospital (JUH)) and laboratory standard strains (American Type Culture Collection (ATCC) and institutional stocks) were used in this study (Table 1). Species identification and antimicrobial resistance testing of the clinical isolates were routinely carried out using VITEK 2 (bioMèrieux, Marcy-l’Étoile, France). The minimal inhibitory concentrations (MICs) of the antibiotics for the laboratory strains were determined by the microdilution technique in accordance with European Antimicrobial Susceptibility Testing Committee (EUCAST) standards [ISO 20776-1:2019] by using a cation-adjusted Mueller Hinton (MH) broth. All isolates were stored in 10% (V/V) glycerol stocks at −80°C and freshly struck on blood agar plates (BD Diagnostics, Heidelberg, Germany) and grown overnight at 37°C before use and adjusted to 0.5 McFarland in an MH medium (Roth GmbH, Karlsruhe, Germany).

**TABLE 1:** Bacterial strains used in this study.

Species	Strain	Sources and comments
*K. pneumoniae*	ATCC 700603	Reference strain
*K. pneumoniae*	Ks 217	Rectal screening swab, ESBL^a^
*K. pneumoniae*	Ks 1415	Rectal screening swab, ESBL
*K. pneumoniae*	Ks 1030	Rectal screening swab, resistant against fluoroquinolones
*A. baumannii*	IIMK 450	Laboratory stock
*A. baumannii*	IIMK 451	Laboratory stock
*A. baumannii*	Bk 05113	Blood culture, unknown primary focus, resistant against carbapenems, cephalosporins, and fluoroquinolones
*A. baumannii*	Va 39036/1	Liquor, resistant against carbapenems, cephalosporins, and fluoroquinolones
*P. aeruginosa*	ATCC 27853	Reference strain
*P. aeruginosa*	IIMK 403	Laboratory stock
*P. aeruginosa*	PA 3	Sputum of patient with CF^b^
*P. aeruginosa*	Va 36580/4	Sputum of patient with, mucoidal growth pattern
*B. cenocepacia*	ATCC 25416	Reference strain
*B. cenocepacia*	BC 1	Sputum of patient with CF
*B. cenocepacia*	BC 2	Sputum of patient with CF
*B. cenocepacia*	Va 3003/3	Sputum of patient with CF
*S. aureus*	ATCC 29213	Reference strain, MRSA^c^
*S. aureus*	Bk 14073	Blood culture, unknown primary focus, MRSA
*S. aureus*	Va 31397	Nasopharyngeal screening swab from a patient with CF, MSSA^d^
*S. aureus*	Va 31505	Conjunctiva swab, conjunctivitis, MSSA
*E. faecalis*	ATCC 29212	Reference strain
*E. faecalis*	Bk 8653	Blood culture, unknown primary focus
*E. faecalis*	Bk 848	Blood culture, unknown primary focus
*E. faecalis*	Bk 1653	Blood culture, unknown primary focus
*E. faecium*	Bk 012713	Blood culture, patient with endocarditis
*E. faecium*	Bk 012539	Blood culture, unknown primary focus
*E. faecium*	Bk 013000	Blood culture, unknown primary focus
*E. faecium*	Bk 013041	Blood culture, unknown primary focus

*^a^Extended-spectrum β-lactamase.*

*^b^Cystic fibrosis.*

*^c^Methicillin-resistant S. aureus.*

*^d^Methicillin-sensitive S. aureus.*

### Human Milk Oligosaccharide Activity Against Planktonic Bacteria

From fresh agar plates, a cell suspension was adjusted to 0.5 McFarland in fresh MH broth and further diluted to 1:100 (correlates to approximately 10^5^–10^6^ CFU/ml). One hundred microliters of the bacterial suspensions was mixed with 100 μl of HMOs or lactose per well in 96-well plates (Greiner Bio-One GmbH, Frickenhausen, Germany) to obtain final concentrations of 0, 5, 10, 20, 50, and 100 mg/ml of HMOs in MH broth or 0, 4, 8, 16, 40, and 80 mg/ml of lactose and incubated at 37°C for 24 h without shaking. As control, the biofilms were treated in the same manner by MH broth only (0 mg/ml). The supernatant was serially diluted in a logarithmic manner up to 10^–8^ in sterile saline (9 g/L NaCl). Subsequently, 10 μl of the dilutions was dropped on non-selective MH-agar plates and incubated overnight at 37°C. The colony-forming units (CFU) per ml were assessed from two to three countable droplets. Each experiment was independently performed in triplicate.

### Determination of the Antibiofilm Activity of the Human Milk Oligosaccharides

For biofilm formation, overnight cultures were diluted to 0.5 McFarland (correlates to 10^7^–10^8^ CFU/ml) in fresh MH broth, and 200 μl of the bacterial suspensions was applied per well into 96-well plates and incubated for 48 h without shaking at 37°C. The supernatants were removed, and the biofilms were carefully washed with 200 μl sterile saline per well. The biofilms were treated for 24 h with 200 μl per well of serially diluted HMOs (5, 10, 20, 50, and 100 mg/ml) or lactose (4, 8, 16, 40, and 80 mg/ml) or 1:2 diluted HMOs in MH broth. As control, the biofilms were treated in the same manner by MH broth only (0 mg/ml). The concentration was not assessed after size-exclusion chromatography (SEC), see below). After treatment, the supernatants were removed, and the biofilms were carefully washed twice with 200 μl sterile saline. The biofilms were resuspended in 200 μl sterile saline by scraping them from the cavities and vortexing. After a serial dilution in a logarithmic manner up to 10^–8^, 10 μl of the dilutions were dropped on MH-agar plates, and the CFU/ml was determined after overnight incubation at 37°C from the countable droplets. Each experiment was independently performed in triplicate.

### Live/Dead Staining of the Biofilms and Confocal Laser Scanning Microscopy Image Acquisition

These experiments were performed only with the selected strains *K. pneumoniae* ATCC 700603, *A. baumannii* IIMK450, *P. aeruginosa* PA 03, *B. cenocepacia* BC 1, *S. aureus* ATCC 29213, *E. faecalis* ATCC 29212, and *E. faecium* Bk 013000. Biofilm formation was performed as previously described but in lumox™ 96-well plates for microscopic analysis (Greiner Bio-One GmbH, Frickenhausen, Germany) in duplicate. The supernatants were removed, and the biofilms were carefully washed with 200 μl sterile saline per well. The biofilms were treated with 200 μl of the HMOs (0, 5, 10, and 20 mg/ml in MH broth) for 24 h. After treatment, the supernatants were removed, and the biofilms were washed twice with 200 μl sterile saline and stained with the LIVE/DEAD BacLight Bacterial Viability Kit for microscopy (Life Technologies GmbH, Darmstadt, Germany) according to the manufacturer’s protocol. Stained biofilms were analyzed under vital conditions using the inverse CLSM LSM780 and the × 40 air objective (both Carl Zeiss AG, Germany) at 488 nm excitation by the argon laser line. An area of approximately 200 μm (X) × 200 μm (Y) was screened in 1 μm Z-intervals (Z-stack) at green (emission 522 nm) and red (emission 635 nm) channels. The pinhole was adjusted to approximately 1 μm.

### Size-Exclusion Chromatography Fractionation of the Human Milk Oligosaccharides

The ÄKTA chromatography system (GE Healthcare, Chicago, United States) was used for the separation of the saccharides by SEC. A P-2 Bio-Gel (Bio-Rad Laboratories, Hercules, United States) column (5 × 175 mm) was manually prepared according to the manufacturer’s instruction. The flow rate was set to 0.1 ml/min, and deionized and degassed water was used for elution. Commercially available D-glucose and D-lactose monohydrate (both Merck KGaA Darmstadt, Germany), as well as the pentose verbascose (O-VER) (Megazyme Inc., Bray, Ireland), were used as standards. The saccharides were prepared as 20 mg/ml solutions in deionized water, and 200 μl was used for SEC separation. The lactase F-digested HMOs were resuspended in 100 μl deionized water and separated. Fractions of 200 μl were collected.

### Thin-Layer Chromatography

The chromatographic fractions were analyzed on TLC silica gel 60 F254 plates (Supelco, Bellefonte, United States). Briefly, 2.5 μl (in 0.5 steps) of fractions F6–F16 and 1 μl each of glucose and lactose (each 20 mg/ml) standards were applied to a silica gel plate. After drying at room temperature, the plate was set into the TLC chamber pre-equilibrated with the liquid phase, a mixture of butanol–isopropanol–water in the volume ratio 3:12:6. The chromatography was performed for 1.5 h, and the plates were dried under the chemical hood overnight. Thereafter, the plates were briefly rinsed in orcinol staining solution (80 mg orcinol monohydrate in 160 ml acetone and 8 ml concentrated sulfuric acid) and then baked at 100°C to stain the sugars.

### Orbitrap Measurements (Ultra-High-Performance Liquid Chromatography/High-Resolution Mass Spectrometry)

Ultra-High-Performance Liquid Chromatography (UHPLC) coupled with High-Resolution Mass Spectrometry (HRMS) was carried out using the UltiMate UHPLC system (all Thermo Fischer Scientific, Bremen, Germany), equipped with an UltiMate HPG-3400 RS binary pump, WPS-3000 autosampler (set to 10°C) with a 25 μl injection syringe, and a 100 μl sample loop. The Hypercarb^®^ column (100 × 2.1 mm; 3 μm) (Thermo Fischer Scientific) was kept at 25°C within the column compartment TCC-3200. Eluent A was water with 5 mM NH_4_OAc buffer (adjusted to pH 9.6) supplemented with 2% acetonitrile. Eluent B was 80% acetonitrile in 5 mM NH_4_OAc buffer (adjusted to pH 9.6). Chromatographic separation was performed by applying a constant flow rate of 0.22 ml/min and a two-step gradient: min 0.2–5 from 0 to 10% eluent B, min 5–20–100% eluent B, min 20–22 constant at 100% eluent B. The HMO fractions were used in a native non-derivatized form. Column recovery was performed by quickly changing (30 s) to 100% eluent A that was run for an additional 3.5 min.

Mass spectra were recorded with a Q Exactive Plus Orbitrap (Thermo Fischer Scientific) MS coupled to a heated electrospray source (HESI). Column flow was switched at 0.5 min from waste to the MS and at 23.5 min again back to the waste, to prevent source contamination. For monitoring, a full-scan mode was selected with the following parameters: polarity: positive; scan range: 200–2,000 *m/z*; resolution: 280,000; AGC target: 3 × 10^6^; and maximum IT: 100 ms. Additionally, a data-dependent MS^2^ mode was executed: scan range: 200–2,000 *m*/*z*; resolution: 35,000; AGC target: 1 × 10^5^; maximum IT: 500 ms; loop count: 2; TopN: 2; isolation window: 0.5 *m*/*z*; no fixed first mass; stepped NCE 15, 30, and 45; exclude isotopes: on; and dynamic exclusion: 10 s. General settings were as follows: sheath gas flow rate: 60; auxiliary gas flow rate: 20; sweep gas flow rate: 5; spray voltage: 2.5 kV; capillary temperature: 360°C; S-lens RF level: 50; vaporizer temperature: auxiliary gas heater temperature: 400°C; and acquisition time frame: 0–24 min.

### Fucosyllactose and Other Compounds

Two isomeric forms of Fucosyllactose (FL) (2′-FL and 3′-FL) were purchased in lyophilized form from Biosynth Carbosynth (Thal, Switzerland). The vials were completely resuspended in 1 ml deionized water to obtain a 10 mg/ml stock solution and stored at 4°C.

Other compounds (if not specified) were purchased from Carl Roth GmbH or Sigma Aldrich.

### Data Analysis and Statistics

All statistics and diagrams of the saccharide activities assessed as CFU/ml were performed using GraphPad Prism version 6.0 for Windows (GraphPad Software, La Jolla, CA, United States^1^). To compare the activities of the HMO fractions, the Mann–Whitney *U*-test for non-parametric samples with a two-tailed confidence interval of 95% was used. Differences with *P* < 0.05 were considered statistically significant. The CLSM biofilm data were visualized by ZEN 9.0 Black software (Carl Zeiss AG, Jena, Germany), and the figures were prepared by CorelDRAW Graphics Suite X5 (Corel, Ottawa, Canada). The MS data were analyzed by Xcalibur 4.3 software (Thermo Fischer Scientific).

## Results

### Antimicrobial Activity of the Total Human Milk Oligosaccharides

The total HMO fractions from pooled milk were investigated for their antimicrobial activity against different species and strains (Table 1). For comparison, the equivalent concentrations of lactose, which make up approximately 80% of the saccharides in milk, were tested in the same manner. The concentration-dependent effects were assessed based on the treatment with MH broth only (0 mg/ml values).

**FIGURE 1:**
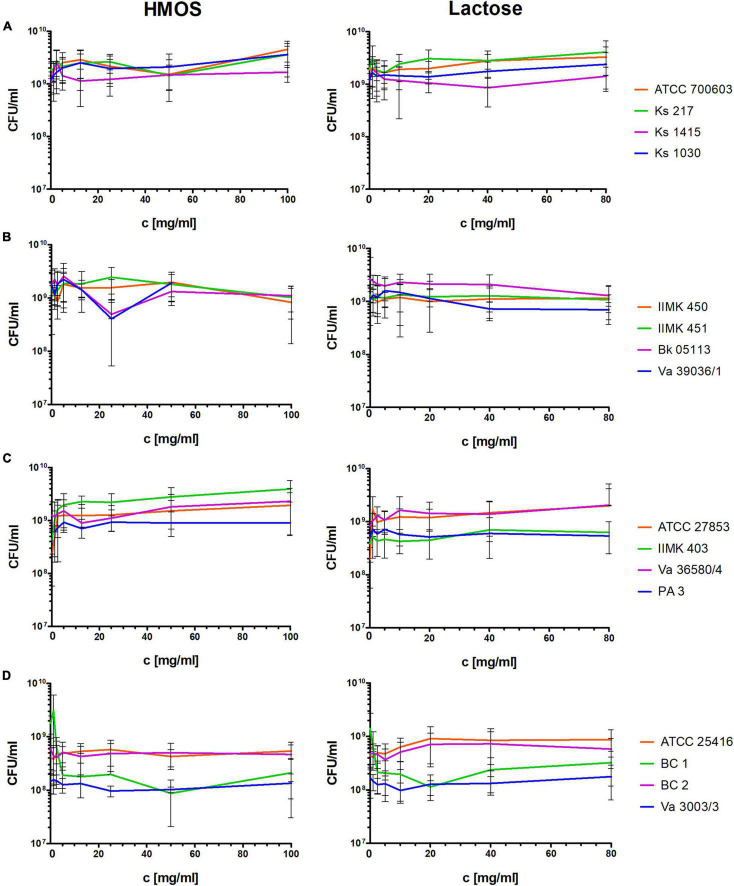
Effects of total HMOs and lactose on laboratory and clinical strains of different Gram-negative species after 24 h of growing under planktonic conditions (assuming that lactose makes up to 80% of the HMOs). **(A)** *K. pneumoniae*, **(B)** *A. baumannii*, **(C)** *P. aeruginosa*, **(D)** *B. cenocepacia*.

**FIGURE 2:**
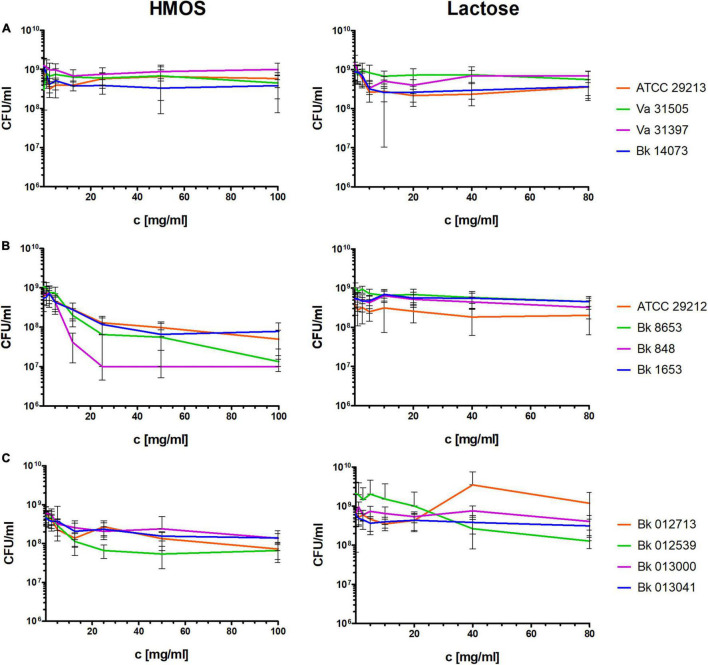
Effects of total HMOs and lactose on laboratory and clinical strains of different Gram-positive species after 24 h of growing under planktonic conditions (assuming that lactose makes up 80% of the HMOs). **(A)** *S. aureus*, **(B)** *E. faecalis*, **(C)** *E. faecium.*

Neither the HMOs nor the lactose showed inhibitory effects on the tested Gram-negative species growing under planktonic conditions (Figure 1). The tested *S. aureus* strains also showed no visible differences in the CFU counts after 24 h HMO or lactose treatment (Figure 2A). In contrast, the CFU count was visibly reduced by the HMOs in a concentration-dependent manner for enterococci (Figures 2B,C), particularly for *E. faecalis*. The effect strength was strain dependent, and the highest reduction (> 2 log-magnitudes) was achieved in strain Bk 848 (Figure 2B).

### Effects Against Mature Biofilm

The effect of the pooled HMOs on mature biofilms was investigated by treating 48 h-grown biofilms for a further 24 h with different HMO or lactose concentrations. Here, too, it was assumed that lactose makes up 80% of the total HMOs.

**FIGURE 3:**
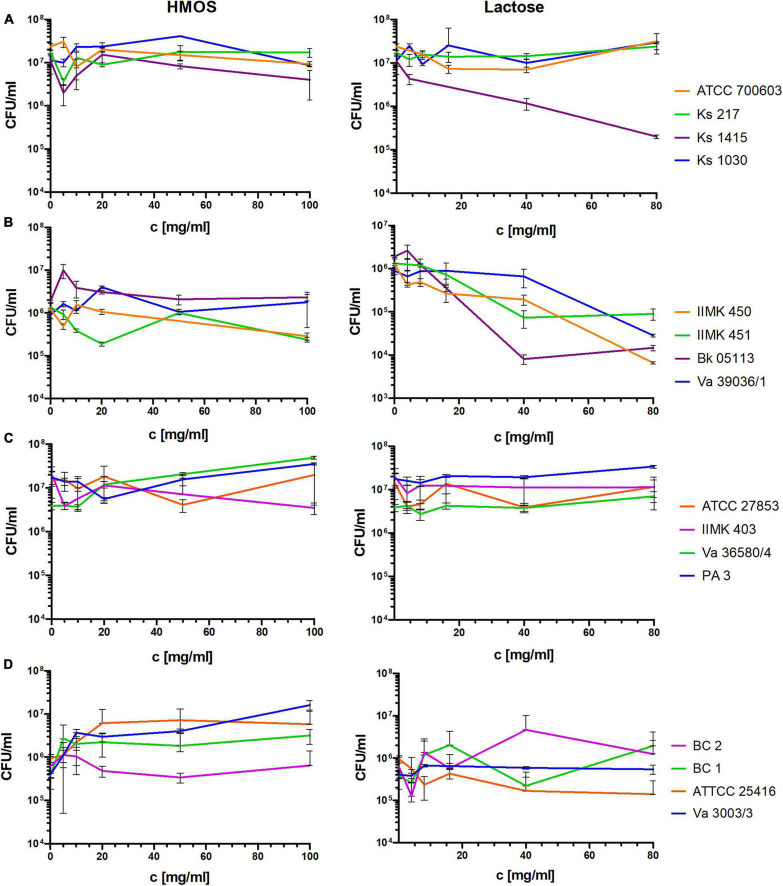
Determination of the viable cell fraction (as CFUs/ml) in biofilms of laboratory and clinical strains of different Gram-negative species after treatment with the total HMOs and lactose in corresponding concentrations (assuming that lactose makes up to 80% of the HMOs) for 24 h. **(A)** *K. pneumoniae*, **(B)** *A. baumannii*, **(C)** *P. aeruginosa*, **(D)** *B. cenocepacia*.

In general, the HMOs showed almost no activity against biofilms of Gram-negative species (Figure 3). A slightly reduced number of viable cells was observed at concentrations below 20 mg/ml in Gram-negative species that, however, did not reach 1-log-level reduction. The effects on *B. cenocepacia* showed more strain-specific variance that might result from the higher genetic variability within this species. On *A. baumannii*, the lactose showed some bactericidal and concentration-dependent effects on the biofilms, reducing the viable cells by 2-log magnitudes (Figure 3B), which might be the result of the unfavorable carbon source for this non-fermenting species. On the other hand, lactose showed no specific effects on *P. aeruginosa* or *B. cenocepacia* (Figures 3C,D), both representing also non-fermenting species. Thus, the observed effect of lactose on *A. baumannii* was elusive.

**FIGURE 4:**
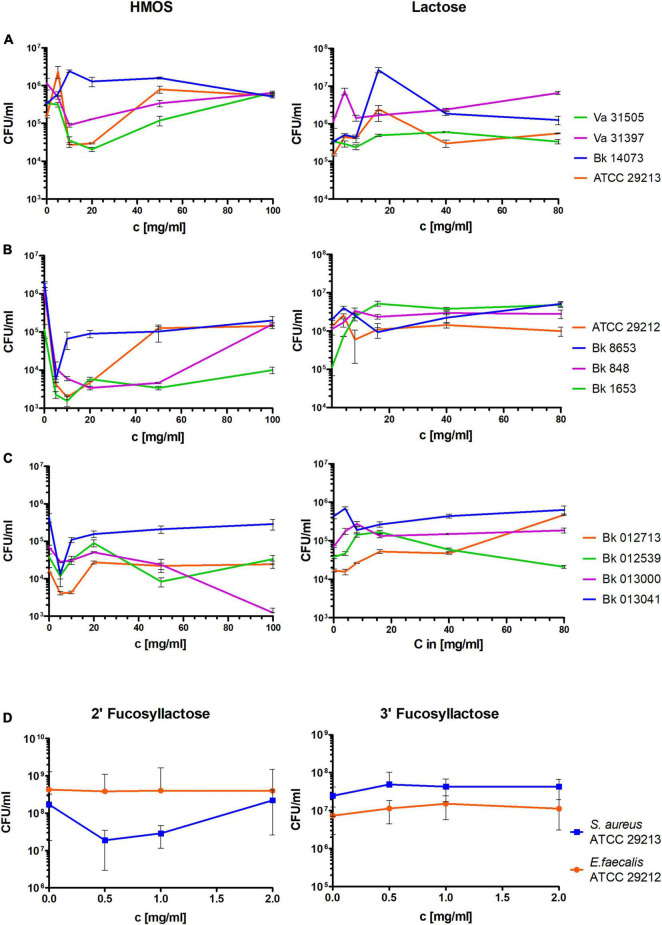
Determination of the viable cell fraction (as CFUs/ml) in biofilms of laboratory standard and clinical strains of different Gram-positive species. **(A–C)** After treatment with the total HMOs and lactose in corresponding concentrations (assuming that lactose makes up 80% of the HMOs) for 24 h. **(A)** *S. aureus*, **(B)** *E. faecalis*, **(C)** *E. faecium*. **(D)** Activities of the FL isomers 2′-FL and 3′-FL against biofilms of *S. aureus* and *E. faecalis* expressed as CFU/ml after treatment. Data of three independent experiments performed in triplicate are given as mean and standard deviation (SD).

Like the planktonic-grown Gram-positive bacteria, lactose did not considerably influence the viable cell numbers, while the HMOs showed a visible reduction of the viable cells of the biofilms by up to a few log levels (Figures 4A–C), particularly in *S. aureus* and *E. faecalis* (Figures 4A,B, respectively). Again, isolate-specific variations in sensitivity were observed, although sensitivity could not be derived directly from the planktonic experiments. Thus, in addition to Bk 848, the biofilms of *E. faecalis* ATCC 29212 and Bk 1653 were also reduced by nearly 3-log levels.

At HMO concentrations higher than 20 mg/ml, the bactericidal effect was abolished in almost all Gram-positive species and isolates. It is likely that at higher saccharide concentrations, lactose and some of the oligosaccharides might enhance growth as nutrients.

### Biofilm Activity in Microscopic Evaluation

**FIGURE 5:**
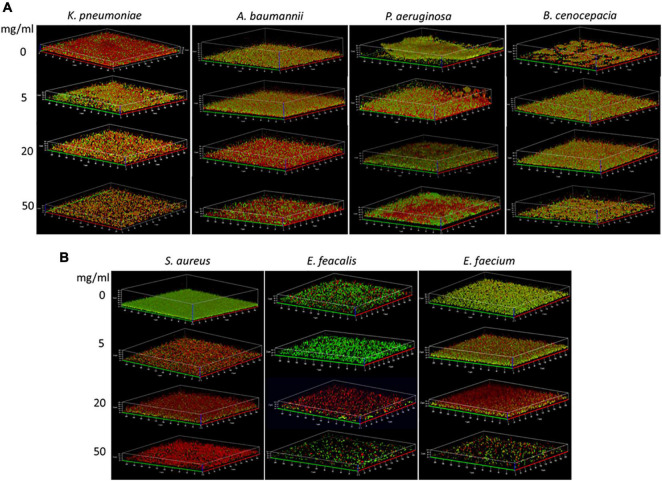
Exemplary 3D images of the CLSM of untreated (0 mg/ml) and HMO-treated (5, 20, and 50 mg/ml) biofilms. **(A)** Gram-negative biofilms of *K. pneumoniae* ATCC 700603, *A. baumannii* IIMK450, *P. aeruginosa* PA 03, and *B. cenocepacia* BC 1. **(B)** Gram-positive biofilms of *S. aureus* ATCC 29213, *E. faecalis* ATCC 29212, and *E. faecium* Bk 013000. Sections of 212 μm × 212 μm (X/Y axes) were screened, and the *Z*-size varied depending on the biofilm thickness. Ticks on the *Z*-axis for *K. pneumoniae* indicate 4 μm, and for the other strains 5 μm. The biofilms were fluorescently stained by SYTO 9 (live, green) and propidium iodide (dead, red) before imaging.

The effect of the HMOs (at 5, 10, and 20 mg/ml) on biofilms was also analyzed by CLSM after live (green)/dead (red) staining and compared to that on MH-only-treated (0 mg/ml) biofilms. Accordingly, in the CFU assessment, the Gram-negative bacteria (*K. pneumoniae* ATCC 700603, *A. baumannii* IIMK450, *P. aeruginosa* PA 03, and *B. cenocepacia* BC 1) showed less sensitivity to the HMOs (Figure 5A). In general, the biofilms of the Gram-negative species showed a higher number of red-stained cells also in the controls (treatment only with saline); thus, it was more difficult to interpret the number of dead cells by purely visual evaluation. The number of red-strained cells seemed to increase while the biofilm size (biomass) decreased in an HMO-concentration-dependent manner in all tested Gram-negative species. This reduced biofilm size agrees with the observed slightly reduced CFU/ml at HMO concentrations below 20–50 mg/ml, but because no strong reduction of the CFU/ml was determined, the red staining seemed not to correspond to cell death in the Gram-negative species. There might be two reasons for this observation: (i) the HMOs might increase the susceptibility to the red dye by interaction with membranes, increasing their permeability; (ii) or the external DNA content of the Gram-negative species led to overlapping staining of the outer layers of the bacteria.

In the Gram-positive species (*S. aureus* ATCC 29213, *E. faecalis* ATCC 29212, and *E. faecium* Bk 013000), the HMO effect on the biofilms was more pronounced (Figure 5B). The *S. aureus* controls were stained intensively green, and the cells turned red in an HMO-concentration-dependent manner while the biofilm size was not visibly reduced. Considering the strong reduction in the CFU/ml, the red staining indicated cell death in *S. aureus* biofilms. Similarly, the red cell fraction increased in *E. faecium* and *E. faecalis* biofilms up to an HMO concentration of 20 mg/ml, but at 50 mg/ml, the biofilms became thin and holey. This indicates that the HMOs not only killed the cells but also led to cell lysis of the enterococci. However, this was not proved by additional experiments and might result from increased release of the cells into the planktonic phase due to matrix modulation by HMOs.

### Evaluation of the Active Human Milk Oligosaccharide Fractions

To decide, after SEC, which fraction should be investigated on the biofilms, the standard oligosaccharides were fractioned by SEC, and their presence in specific fractions was investigated by TLC and staining. The glucose (the monosaccharide) was identified in fractions 12–14 (maximum in 13) and lactose (disaccharide) in fractions 11 –13 (maximum in 12). The pentose O-VER was identified in fractions 10 and 11 (maximum in 10).

From lactase F-digested HMOs, 100 mg was separated by SEC per run. Fractions 6–14 (each 200 μl) were diluted 1:2 in deionized water and tested for their antibiofilm activity against matured biofilms of *S. aureus* ATCC 29213 for 24 h. MH broth was used as control; additionally, 100 mg/ml glucose and lactose was applied to compare the effects of possible residues. The effect of galactose was not tested. Before washing of the biofilms and CFU/ml assessment after treatment, the cells released from the biofilms into the planktonic phase were harvested and serially diluted, and the CFUs/ml were assessed as well.

**FIGURE 6:**
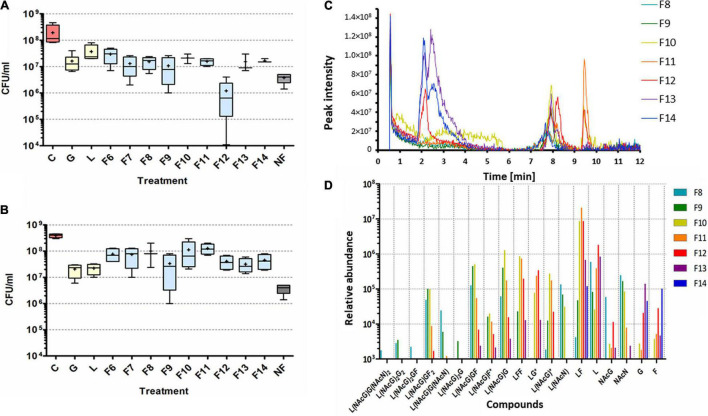
Analysis of UHPLC/HRMS fractionated HMOs. Activities of the HMO fractions against biofilms **(A)** and planktonic bacteria **(B)** of *S. aureus* expressed as CFU/ml after treatment. The X-axis indicates the different treatments. C, control, no treatment (red); G, glucose; L, lactose (both yellow). The SEC fractions used for the treatment are indicated with blue bars according to their number. *NF*, non-fractionated HMOs (gray). = = = The boxes represent the range of the data (max to min), the bars show the standard deviation (SD), the lines in the boxes indicate the median, and the crosses indicate the averaged values. **(C)** UHPLC sensorgrams of the HMO fractions F8–F14. The sensorgrams were normalized to the control (phosphate-buffered saline). **(D)** Relative abundance of the oligosaccharides detected in the SEC fractions F8–F14 by HRMS. Only signals above a relative abundance of 103 were shown. The saccharides or saccharide moieties were calculated based on the *m*/*z* signals (mass/charge) of the H^+^, Na^+^, or K^+^ adducts; the stereochemistry was not assessed. The compounds are given as moieties as follows: F, fucose; G, glucose or galactose; NAcG, *N*-acetyl glucosamine; NAcN, *N*-acetylneuraminic acid (sialic acid); and L, lactose; * indicates possible cleavage products.

Compared to glucose and lactose, fraction 12 showed the highest antibiofilm activity, reducing the viable bacteria of the biofilms by up to 2-log magnitudes in a significant (*P* = 0.002) manner (Figure 6A). Compared to lactose, fractions 11 and 13 also showed significant reduction (*P* = 0.027 and 0.044, respectively). Since monosaccharides and disaccharides were eluted in this fraction in the preliminary tests, this indicates that the active ingredients must have a low molecular weight with few saccharide moieties. No significant effects of the fractionated HMOs were observed in the cells of the planktonic phase (Figure 6B).

Fractions 8–14 were further analyzed by UHPLC/HRMS to assess the most abundant saccharides in these fractions. All identified compounds are listed in the Supplementary Material. In the UHPLC, a peak that was unique in fractions 11–13, but highest in fraction 12 (Figure 6C), was visible at 8.02 min (retention time), which might correspond to the active compound as identified in the biofilm assays. However, in fraction 12, only galactosyl lactose (GL) as a possible active compound was identified but at low relative abundance (3.4 × 10^5^) compared to other compounds (Figure 6D). Lactose was generally the second-highest abundant compound and was found in all fractions. In fractions 13–14, lactose might result from still incomplete digestion but with decreasing fraction number (higher molecular weights); the source must be rather a defragmentation of the compounds by the ionization energy of the lactase F digestion. Lactase F hydrolyzes the terminal non-reductive galactose residues of β-D-galactosides, and thus, some of the HMOs might be possible substrates. It could be previously shown that lactase F partially digested lacto-*N*-tetraoses (LNT) but not the fucosylated or sialylated HMOs resulting in lacto-N-triose as a new digestion product (Gnoth et al., 2000). Thus, the observed trioses [LG* and L(NAcN)*] in the present study might correspond to lactase digestion of LNT [L(NAcG)G in Figure 6D].

FL isomers showed the highest abundance and were identified in fractions 10–12 (with > 10^7^ being highest in fraction 11). FL might be fucosylated either at 2′-C of galactose or at 3′-C of glucose. Difucosyllactose (LFF) was also present in lightly elevated concentrations in fraction 10 (8.6 × 10^5^) to 12 (1.9 × 10^5^).

No sialylated HMOs were identified in the tested fractions; however, an *N*-acetylneuraminic acid residue was identified in all fractions except fractions 12 and 14 at very low abundance; thus, it cannot be excluded that such compounds were present in very low concentrations but were defragmented during the analysis.

### Antibiofilm Activity of Fucosyllactose

In the biofilm-activity assays, the lower fractions containing higher saccharides did not exhibit visible antibiofilm activity. Thus, it was assumed that the active compound must be a smaller HMO. As FL represented the main compound identified in fractions 11–13, both commercially available isomers, 2′-FL or 3′-FL, were here analyzed on 48 h-grown biofilms of *S. aureus* and *E. faecalis* (both ATCC strains) for their antibiofilm activities (Figures 4C,D). However, both isomers showed almost no effects on the biofilms of both species. 2′-FL weakly reduced viable cells in *S. aureus* biofilms (approximately 1 log) at lower concentrations, but the effect was abolished at 2 mg/ml, indicating that FL was not the searched active compound responsible for the antibacterial effect against these Gram-positive bacteria.

## Discussion

A preliminary study demonstrated that the intestinal flora of breastfed infants changes within the first weeks of life from non-HMO-consuming potentially pathogen genera, mainly *Streptococcaceae* but also *Enterobacteriaceae* and *Staphylococcaceae*, to HMO-consuming and beneficial ones, particularly *Bacteroidaceae* and *Bifidobacteriaceae* (De Leoz et al., 2015; Rosenberg et al., 2019; Zhou et al., 2020).

The present work revealed antimicrobial and antibiofilm activities of pooled and fractionated HMOs against Gram-positive bacteria. On the planktonic level, the activity was strongest against *E. faecalis* followed by *E. faecium*, while on the biofilm level *S. aureus* cell counts could also be reduced visibly. These results are generally in accordance with previous studies, where Ackerman et al. (2018) showed antibacterial and antibiofilm properties against *S. aureus* and *S. agalactiae* but only weak effects on planktonic *A. baumannii* for the HMOs obtained from selected donors. Visible activity against Gram-negative species was not detected under the selected conditions, confirming a rather Gram-positive mode of action of the HMOs.

In other studies, a strong variation in the antimicrobial activity between the donors but also between different strains was observed, indicating the highly individual composition of the HMOs in the donors. Therefore, we used pooled HMOs from nine donors to identify the active compounds by fractionation of the pooled HMOs and by testing fractions 6–14 against planktonic and biofilm-grown *S. aureus*. The highest activity determined in fraction 12 correlated to low-molecular-mass HMOs with less than four saccharide moieties. Because lactose could be excluded as an active compound, the next logical conclusion was FL (2′-FL) as an active component. Recently, the shift in microbiota composition to probiotic bifidobacteria and other butyrate-producing bacteria for 2′-FL in an *in vitro* gastrointestinal (GI) fermenter model was demonstrated (van den Abbeele et al., 2019). There, a microbiome obtained from an infant’s stool (three donors) was first established and treated for 3 weeks with 2 mg/ml 2′-FL that corresponded to the highest concentration in our study to treat the biofilms of *S. aureus* and *E. faecalis*. However, we did not observe any notable antibacterial effect of 2′-FL on these two species, nor of the 3′-FL, its isomeric form. The beneficial or prebiotic effects of 2′-FL are not due to antimicrobial activity against human pathogenic bacteria. Most likely, the main role of 2′-FL is immunomodulation, as demonstrated previously for invasive *Escherichia coli* in intestinal epithelial cells, where 2′-FL was shown to inhibit LPS-mediated inflammation by blocking the CD14/IL8 pathway (He et al., 2016). Both FL isomers have also been shown to reduce the binding of different Gram-negative species (including *E. coli* and *P. aeruginosa*) to intestinal and respiratory epithelial cells (Weichert et al., 2013). These might be associated with an interaction with pathogenic surface structures, such as lectins (Chemani et al., 2009), that are also produced by immune cells and are known HMO receptors (reviewed in Rousseaux et al., 2021).

Other low-molecular-mass HMOs that could be considered as anti-Gram-positive active compounds in the present study might be GL and/or sialyllactoses (SL). The first was identified in fraction 12, but not the second. The GL is commercially available but was not investigated in this study, as the oligosaccharide did not contain any specific functional group (fucosyl or sialyl) and was represented in relatively low concentration in the HMO pool. However, the anti-inflammatory effect of different GL isomers was demonstrated previously (Newburg et al., 2016), indicating bioactive features.

Although no efficacy against Gram-negative bacteria was observed, it would be interesting to see whether HMOs (as a pool or as individual components) improve the efficacy of antibiotics, especially against biofilms. A synergistic interaction has been shown for aminoglycosides, lincosamides, macrolides, and tetracyclines against *S. agalactiae* and for aminoglycosides against *S. aureus* and *A. baumannii* (Craft et al., 2018). However, synergism in biofilms was poorly tested so far but might open new opportunities to restore antibiotic activity and reduce uncontrolled inflammation in the treatment of biofilm-associated infections.

The biological significance of these findings may lie in the prevention of skin infections in the mothers’ breast and the infants’ nasopharynx, as *S. aureus* is commonly colonizing the skin and mucous membranes and can be easily transmitted during breastfeeding. The natural habitat of enterococci is the GI tract, and they belong to the first lactic bacteria colonizing the neonatal GI tract (Fanaro et al., 2003). Transmissions to skin and mucosa by fecal smear are common. HMOs may also balance the portion of enterococci in the GI tract of infants. It would certainly be interesting to investigate the bioactivity of HMOs also against other human pathogens.

## Data Availability Statement

The original contributions presented in the study are included in the article/Supplementary Material, further inquiries can be directed to the corresponding author/s.

## Ethics Statement

The studies involving human participants were reviewed and approved by the Ethics Committee of Medical University of Warsaw, Warsaw, Poland (registry number AKBE/180/2018). The patients/participants provided their written informed consent to participate in this study.

## Author Contributions

GO, SJ, and OM contributed to conception of the study. GO, SJ, OM, and NU organized the methodology. SJ, KS, MGM, NU, TT, RS, TK, and OM performed the investigation. SJ, KS, OM, NU, and RS provided the data analysis. GO, MWP, and AW were responsible for the resources. OM and SJ wrote the original draft. OM, SJ, NU, and MWP reviewed and edited the manuscript. OM made the visualization. GO and OM provided supervision and project administration. All authors contributed to manuscript revision and read and approved the submitted version.

## Conflict of Interest

The authors declare that the research was conducted in the absence of any commercial or financial relationships that could be construed as a potential conflict of interest.

## Publisher’s Note

All claims expressed in this article are solely those of the authors and do not necessarily represent those of their affiliated organizations, or those of the publisher, the editors and the reviewers. Any product that may be evaluated in this article, or claim that may be made by its manufacturer, is not guaranteed or endorsed by the publisher.

## Footnotes

1
www.graphpad.com
